# Patterns of Injuries Among Children Diagnosed With Attention Deficit Hyperactivity Disorder in Aseer Region, Southwestern Saudi Arabia

**DOI:** 10.7759/cureus.17396

**Published:** 2021-08-23

**Authors:** Mahdi M Alqarni, Ayed A Shati, Mohammed Z Alassiry, Waddah M. A Asiri, Saeed S Alqahtani, Ahmed S ALZomia, Naif A Mahnashi, Mushary S Alqahtani, Faisal S Alamri

**Affiliations:** 1 Pediatric Orthopedics, Abha Maternity and Children Hospital, Abha, SAU; 2 Child Health, College of Medicine, King Khalid University, Abha, SAU; 3 Psychiatry, Abha Psychiatry Hospital, Abha, SAU; 4 Psychiatry, Department of Medicine, College of Medicine, King Khalid University, Abha, SAU; 5 Medicine, College of Medicine, King Khalid University, Abha, SAU; 6 Medicine, College of Medicine, Najran University, Najran, SAU

**Keywords:** attention deficit hyperactivity disorder, injury, trauma, children, pattern

## Abstract

Background

Globally, attention deficit hyperactivity disorder (ADHD) is the most common neurobehavioral disorder that affects children. In 2011, there was an ADHD diagnosis prevalence of around 8% among children (4-17 years) in the US. ADHD-affected children are more prone to physical injuries such as physical trauma, accidental poisoning, burns, etc. This study was aimed to evaluate the association of ADHD with severe injuries, the influence of age and gender on this association, and the impact of ADHD medications on the frequency of such injuries.

Methodology

This study was conducted in three governmental and three private settings in Aseer region. The files of children who were diagnosed with ADHD in the study settings were reviewed for a 12-month time period. Data were extracted from the medical files using a pre-structured data extraction sheet to avoid errors and inter-rater bias. The extracted data included child gender, age, duration of disease, and injury-related data. A brief questionnaire had been applied to mothers regarding mothers' attitudes towards injuries among their children, adherence to medications, as well as the reasons for non-adherence to medications and clinical visits in a non-adherent group during the clinic visit.

Results

One hundred and sixty-three children with a diagnosis of ADHD completed the study. The affected children were aged between two and 15 years (mean: 7.8 ± 2.9 years). An exact of 116 (71.2%) children were males. An exact of 70 (42.9%) affected children had trauma. The most-reported traumas were superficial injuries (84.3%), burns (48.6%), fractures (37.1%), deep injuries (31.4%), and broken or lost teeth (28.6%). About 52% of the children were adherent to medications and their clinical visits. Among the non-adherent group, the most reported reasons were parents’ care and attention (20.5%), followed by the COVID-19 pandemic and delay in visits times (16.7%). Regarding mothers' attitudes towards injuries among children with ADHD, 49.1% of the mothers agreed that there is an association between a child with ADHD and being traumatized while 22.7% said there was no relation.

Conclusions

In our cohort, the majority of the children with ADHD were boys at primary school age. Association of the history of the disease with trauma was not uncommon, and most injuries were not severe, but burns and deep injuries were reported among considered portions.

## Introduction

Attention deficit hyperactivity disorder (ADHD) is a well-known neurobehavioral disorder with childhood-onset and becomes apparent in the preschool and early school years. It is characterized by persistent lack of attention, overactivity, and impulsivity [1]. The three major subtypes of ADHD include: hyperactivity-impulsivity ADHD, inattention ADHD, and combined inattentive/hyperactive-impulsive ADHD (combined ADHD) [1-3]. The Diagnostic and Statistical Manual of Mental Disorders Fourth Edition (DSM-IV) enlists the criteria that are used for ADHD diagnosis [4]. ADHD diagnosis primarily depends on the information collected from the child’s parents, school, and health professionals (if consulted), accompanied by an interview and an examination [5-6].

Previous studies have reported a 4%-12% ADHD incidence among children aged six to 12 years [6-7]. Other studies have reported an ADHD prevalence of 4%-8%, 7.6%-9.5%, 10a%-20%, and 29.7% in the US, Korea, India, and the UAE [7-10]. However, there is limited data with respect to the prevalence and severity of ADHD in Saudi Arabia. Abolfotouh (1997) examined the behavioral aspects of 305 schoolboys with ages between eight and 12 years in Abha, southwestern Saudi Arabia [11]. They reported a 13.4% and 6.9% prevalence of behavioral anomalies and antisocial behavior in their cohort. In a review, Al Haidar (2003) examined 416 case records of children less than 18 years old and reported a 25.5% ADHD prevalence rate, with 12.7% cases suffering from only ADHD and 12.7% suffering from other psychiatric disorders too [12-13]. Al Zaben FN et al. (2018) found an overall ADHD incidence of 5%, with a higher incidence in boys than girls. They reported that the most prevalent subtype of ADHD was combined ADHD (2.7%), followed by hyperactive ADHD and inattentive ADHD (1.2% and 1.1%, respectively) [13].

Previous studies have demonstrated ADHD-affected children are more prone to physical injuries such as physical trauma, accidental poisoning, burns, etc. This study was aimed to evaluate the association of ADHD with severe injuries, the influence of age and gender on this association, and the impact of ADHD medications on the frequency of such injuries.

## Materials and methods

A descriptive cross-sectional study was conducted in Abha Maternity and Children’s Hospital, Khamis Mushait Maternity and Children’s Hospital, Abha Psychiatric Hospital, Hayat National Hospital, Abha Private Hospital, Saudi German Hospital (Aseer), and Daweni Medical Center. Files for children who were diagnosed with ADHD in the study settings were reviewed for a 12-month time period. Data were extracted from the medical files using a pre-structured data extraction sheet to avoid errors and inter-rater bias. Data extracted included child gender, age, and disease duration. Injury history since diagnosis, including the type of injury, frequency of injuries, and required medical care, were also reviewed. A brief questionnaire was applied to mothers regarding mothers' attitudes towards injuries among their children, adherence to medications, as well as the reasons for non-adherence to medications and clinical visits in a non-adherent group during a clinic visit.

Data analysis

The data were statistically analyzed using SPSS version 22 (IBM Corp, Armonk, NY). Two-tailed tests were used for statistical comparisons. Statistical significance was defined by a p-value of < 0.05. We performed descriptive analysis for all variables, including child demographic data, trauma types and frequency, childrens' adherence to treatment, and mothers’ attitude towards disease relation with trauma. The trauma history of children was assessed on the basis of their personal data and adherence to treatment. In addition, the Pearson chi-square test was used to assess the correlations.

## Results

A total of 163 children with a diagnosis of ADHD completed the study. The ages of the affected children ranged from two to 15 years (mean: 7.8 ± 2.9 years). An exact of 116 (71.2%) children were males (Table 1).

**Table 1:** Trauma incidence and pattern among children with ADHD in Aseer region, Saudi Arabia ADHD: attention deficit hyperactivity disorder

Child trauma history	No	%
A child exposed to trauma after diagnosis / last five years		
Yes	70	42.9%
No	93	57.1%
Type of trauma (n=70)		
Superficial injuries	59	84.3%
Burns	34	48.6%
Fractures	26	37.1%
Deep injuries	22	31.4%
Broken/lost tooth	20	28.6%
Eye injury	12	17.1%
Jaw injury	7	10.0%
Anomalies	6	8.6%
Others	5	7.1%
How many times child exposed to trauma (n=70)		
1-3	34	48.6%
4-10	24	34.3%
> 10	12	17.1%
Trauma needed for hospitalization/surgery or any medical intervention (n=70)		
Yes	46	65.7%
No	24	34.3%

Table 2 shows a trauma incidence and pattern among children with ADHD. An exact of 70 (42.9%) affected children had trauma. The most-reported trauma were superficial injuries (84.3%), burns (48.6%), fractures (37.1%), deep injuries (31.4%), broken or lost teeth (28.6%), and eye injuries (17.1%) while anomalies were the least reported trauma (8.6%). Regarding the frequency of getting injury since diagnosis, one to three times were reported by 48.6% of the ADHD affected-children, four to 10 times was recorded among 34.3% of the ADHD children while 17.1% had been injured more than 10 times since diagnosis. Trauma required hospitalization/surgery or any medical intervention among 65.7% of the traumatized children.

**Table 2:** Personal data of children with ADHD in Aseer region, Saudi Arabia ADHD: attention deficit hyperactivity disorder

Personal data	No	%
Age in years		
< 6 Yrs.	40	24.5%
6-9	78	47.9%
10-15	45	27.6%
Gender		
Male	116	71.2%
Female	47	28.8%

Table 3 illustrates clinical care adherence among children with ADHD. An exact 52.1% of the study children were adherent to medications and their clinical visits. Among the non-adherent group, the most reported reasons were parents’ care and attention (20.5%), followed by the COVID-19 pandemic and delay in visits times (16.7%), mothers' perception that their children are good (14.1%), thinking that treatment is ineffective (9%), and being too late (10.3%). An exact 33.1% of the children’s caregivers confirmed that physicians ask about physical trauma during child visits, and 10.4% said that happens sometimes.

**Table 3:** Clinical care adherence among children with ADHD in Aseer region, Saudi Arabia ADHD: attention deficit hyperactivity disorder

Childcare	No	%
Child adherent to medications and clinical visits		
Yes	85	52.1%
No	78	47.9%
If no, mention reasons (n=85)		
No reason	11	14.1%
Parents care and attention	16	20.5%
Covid 19 pandemic	13	16.7%
Child is good	11	14.1%
Treatment is ineffective	7	9.0%
Too late to do	8	10.3%
The financial cost and hospital far away	7	9.0%
Fear of sociality	5	6.4%
The physician asks about any physical trauma during the child visit.		
Yes	54	33.1%
Sometimes	17	10.4%
No	92	56.4%

**Figure 1:**
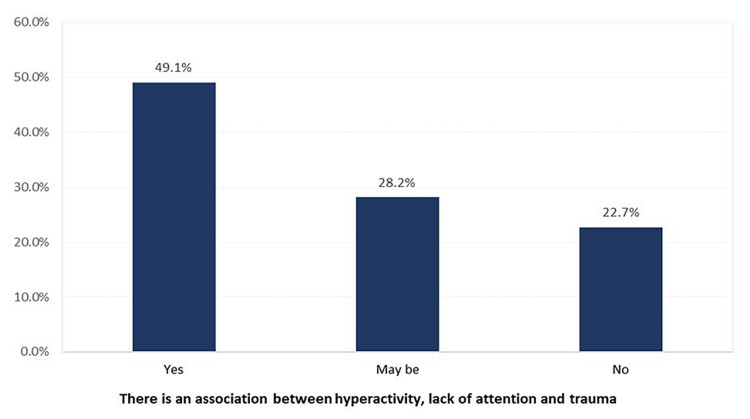
Mothers attitude towards injuries among children with ADHD in Aseer, Saudi Arabia ADHD: attention deficit hyperactivity disorder

Regarding mothers' attitudes towards injuries among children with ADHD (Figure 1), 49.1% of the mothers agreed that there is an association between a child with ADHD and being traumatized while 22.7% said there was no relation.

Table 4 shows the distribution of child trauma, personal data, and medical care adherence. History of trauma was reported among 46.7% of children aged 10-15 years in comparison to 35% of those aged less than six years with no statistical significance (P=0.469). Also, 44.7% of female children had trauma compared to 42.2% of males (P=0.776). Trauma was reported among 48.2% of ADHD children who were adherent to their medication and clinical visits compared to 37.2% of those who did not (P=0.154). Precisely 48.6% of children for mothers who ignore the relation between ADHD and childhood trauma had a history of trauma compared to 46.3% of those who know that relation (P=0.240).

**Table 4:** Distribution of child trauma, personal data, and medical care adherence

Factors	A child exposed to trauma after diagnosis / last five years				P-value
	Yes		No		
	No	%	No	%	
Age in years					.469
< 6 Yrs.	14	35.0%	26	65.0%	
6-9	35	44.9%	43	55.1%	
10-15	21	46.7%	24	53.3%	
Gender					.776
Male	49	42.2%	67	57.8%	
Female	21	44.7%	26	55.3%	
Child adherent to medications and clinical visits					.154
Yes	41	48.2%	44	51.8%	
No	29	37.2%	49	62.8%	
There is an association between hyperactivity, lack of attention, and trauma					.240
Yes	37	46.3%	43	53.8%	
Maybe	15	32.6%	31	67.4%	
No	18	48.6%	19	51.4%	

## Discussion

The current study aimed to identify the relation between ADHD and injuries in children, considering age and sex in Aseer region, Kingdom of Saudi Arabia, and to recognize the most common injures among these spectra. Injuries are deemed a main public health problem, which is the primary cause of mortality among children in the US [14]. Falls, burns, and cut wounds are other types of injuries that cause a perceptible burden globally, leading to disabilities, adverse psychological effects, and death [15-18]. In order to reduce the frequency of injuries, it is necessary to assess the epidemiology of such injuries and determine their risk factors.

ADHD is a psychiatric disorder that exhibits childhood onset. Nonetheless, it is also prevalent among adults, with an incidence rate of up to 5% [19]. There is a semi-consensus regarding that difficulty with continued attention and lack of appropriate response to situations among ADHD-affected individuals. Furthermore, compared to normal individuals, ADHD-affected children exhibit a higher injury risk [20-23].

The current study revealed that nearly three-quarters of the children with ADHD were boys and aged six to nine years. Regarding injury history, less than half of the study children with ADHD had been injured during the last five years. The most reported type of injury was superficial injury, followed by burns and fractures. Deep injuries were one-third of all reported injuries while one-quarter were broken or lost teeth. Among those injured, it happened one to three times among nearly half of them, and two-thirds of the injured children needed hospitalization or medical or surgical management.

Several investigators focused on the association between ADHD and injuries in terms of the impact of ADHD on the frequency of such injuries. Such association might also be dependent on the traumatic brain injuries since previous studies have demonstrated that such brain injuries may lead to ADHD incidence [24-25].

Carla D et al. found a higher incidence of ADHD among boys (66.5%), with prevalence rates of pedestrian- and bicyclist-related injuries of 27.5% and 13.8%, respectively [26]. They also reported a significantly low incidence of self-inflicted injuries (0.1%). They found that a higher frequency of ADHD-affected children was prone to multiple body and head injuries (43% and 41%, respectively). Conversano E et al. assessed the frequency of admittance to the ER on account of ADHD-related injuries and behavior [27]. They reported the admittance of 545 such cases, 251 cases with injuries. In their cohort, 9% of cases visited the ER owing to physical injuries and 10% owing to behavior related to ADHD.

Appropriate treatment and psychological therapy may be effective in reducing the injury- and mortality-related risk among ADHD-affected children, making it possible to reduce the risk of injury and death among children with ADHD. So, the use of adequate ADHD screening techniques is recommended for children with frequent injuries.

## Conclusions

Our results demonstrated that the majority of the children with ADHD were boys at primary school age. History of disease-associated trauma was not uncommon, and most injuries were not severe, but burns and deep injuries were reported among considered portions. Also, the need for medical care due to childhood trauma was considerably reported, which means more care should be paid, and mothers need health education sessions to enhance their awareness regarding how to deal with a child with ADHD and the specific environmental constraints in their home to avoid injuries. Future studies must focus on assessing the impact of early screening for children with ADHD-related behaviors. Such techniques could facilitate early diagnosis, which could, in turn, avoid the development of severe ADHD-related impairments from childhood to adulthood.
